# Efficient TMS-Based Motor Cortex Mapping Using Gaussian Process Active Learning

**DOI:** 10.1109/TNSRE.2021.3105644

**Published:** 2021-08-30

**Authors:** Razieh Faghihpirayesh, Mathew Yarossi, Tales Imbiriba, Dana H. Brooks, Eugene Tunik, Deniz Erdoğmuş

**Affiliations:** Cognitive Systems Laboratory, Electrical and Computer Engineering Department, Northeastern University, Boston, MA 02115 USA; Center for Signal Processing, Reasoning, and Learning (SPIRAL), Electrical and Computer Engineering Department, Northeastern University, Boston, MA 02115 USA; The Movement Neuroscience Laboratory, Department of Physical Therapy Rehabilitation and Movement Science, Northeastern University, Boston, MA 02115 USA.; Cognitive Systems Laboratory, Electrical and Computer Engineering Department, Northeastern University, Boston, MA 02115 USA; Center for Signal Processing, Reasoning, and Learning (SPIRAL), Electrical and Computer Engineering Department, Northeastern University, Boston, MA 02115 USA; Cognitive Systems Laboratory, Electrical and Computer Engineering Department, Northeastern University, Boston, MA 02115 USA; Center for Signal Processing, Reasoning, and Learning (SPIRAL), Electrical and Computer Engineering Department, Northeastern University, Boston, MA 02115 USA; The Movement Neuroscience Laboratory, Department of Physical Therapy Rehabilitation and Movement Science, Northeastern University, Boston, MA 02115 USA.; Cognitive Systems Laboratory, Electrical and Computer Engineering Department, Northeastern University, Boston, MA 02115 USA; Center for Signal Processing, Reasoning, and Learning (SPIRAL), Electrical and Computer Engineering Department, Northeastern University, Boston, MA 02115 USA

**Keywords:** Active learning, entropy, Gaussian process, machine learning, motor cortex, motor evoked potentials, transcranial magnetic stimulation

## Abstract

Transcranial Magnetic Stimulation (TMS) can be used to map cortical motor topography by spatially sampling the sensorimotor cortex while recording Motor Evoked Potentials (MEP) with surface electromyography (EMG). Traditional sampling strategies are time-consuming and inefficient, as they ignore the fact that responsive sites are typically sparse and highly spatially correlated. An alternative approach, commonly employed when TMS mapping is used for presurgical planning, is to leverage the expertise of the coil operator to use MEPs elicited by previous stimuli as feedback to decide which loci to stimulate next. In this paper, we propose to automatically infer optimal future stimulus loci using active learning Gaussian Process-based sampling in place of user expertise. We first compare the user-guided (USRG) method to the traditional grid selection method and randomized sampling to verify that the USRG approach has superior performance. We then compare several novel active Gaussian Process (GP) strategies with the USRG approach. Experimental results using real data show that, as expected, the USRG method is superior to the grid and random approach in both time efficiency and MEP map accuracy. We also found that an active warped GP entropy and a GP random-based strategy performed equally as well as, or even better than, the USRG method. These methods were completely automatic, and succeeded in efficiently sampling the regions in which the MEP response variations are largely confined. This work provides the foundation for highly efficient, fully automatized TMS mapping, especially when considered in the context of advances in robotic coil operation.

## I. Introduction

TMS has become a valuable noninvasive method to map the motor cortical representation of a specific muscle [1]. The basic procedure is to apply TMS pulses to a variety of locations on the scalp overlying the sensorimotor cortex while recording EMGs from target muscle(s), and quantifying the amplitude of resultant MEPs. The resulting “map” of MEP amplitudes as a function of TMS pulse positions describes an estimate of the topographic organization of the muscle(s) under study. The cortical muscle topographies identified by TMS mapping have shown high correlation to the areas identified by other more expensive and burdensome imaging modalities such as fMRI and PET [2]-[4]. Consequently, TMS mapping has become a popular tool for research studies and clinical applications such as tracking cortical reorganization and neurosurgical planning. The current standard technique for mapping involves stimulating with the TMS coil 3-10 times at each site on a regular grid, most commonly consisting of 25-100 points at a 0.5-1 cm spacing. These points are typically identified on the scalp by physically drawing on a swimmer’s cap or using computer-guided navigation systems [5], [6]. Given the large number of points, and a recommended interstimulus time on the order of several seconds, mapping procedures may take as long as one hour to complete. In practice, much of this time is spent recording null responses, since the majority of the stimulation points typically lie outside of the excitable area for the muscle of interest [7]. This time scale prohibits measurement of transient cortical stimulation or learning-induced changes and is not well tolerated by clinical populations. Previous approaches to improving the efficiency of mapping have included stimulating in a spiral pattern by moving radially out from a pre-determined locus or “hotspot” to reduce null stimulation points [8], or by replacing the grid with a cross-like pattern [7] aligned to the precentral gyrus. Recently, a pseudo-random walk sampling pattern combined with interpolation of excitability among the stimulation sites was reported to produce statistically similar outcomes to grid-based approaches with comparatively fewer stimuli [9]-[11].

When TMS mapping is used for surgical planning, the need for greater efficiency has led to an operator dependent (USRG) approach, in which stimulus locations are chosen by the TMS coil operator based on real time feedback of observed MEP responses [12]. The operator is free to increase or decrease stimulation point density at their discretion. This typically reduces the number of null stimulations while simultaneously increasing stimulation density in responsive areas. A number of studies on the use of TMS for preoperative planning using this approach have reported favorable outcomes [12]-[14], including high correlation to “gold standard” maps produced using intracranial electrical stimulation [3], [15].

**Fig. 1.**
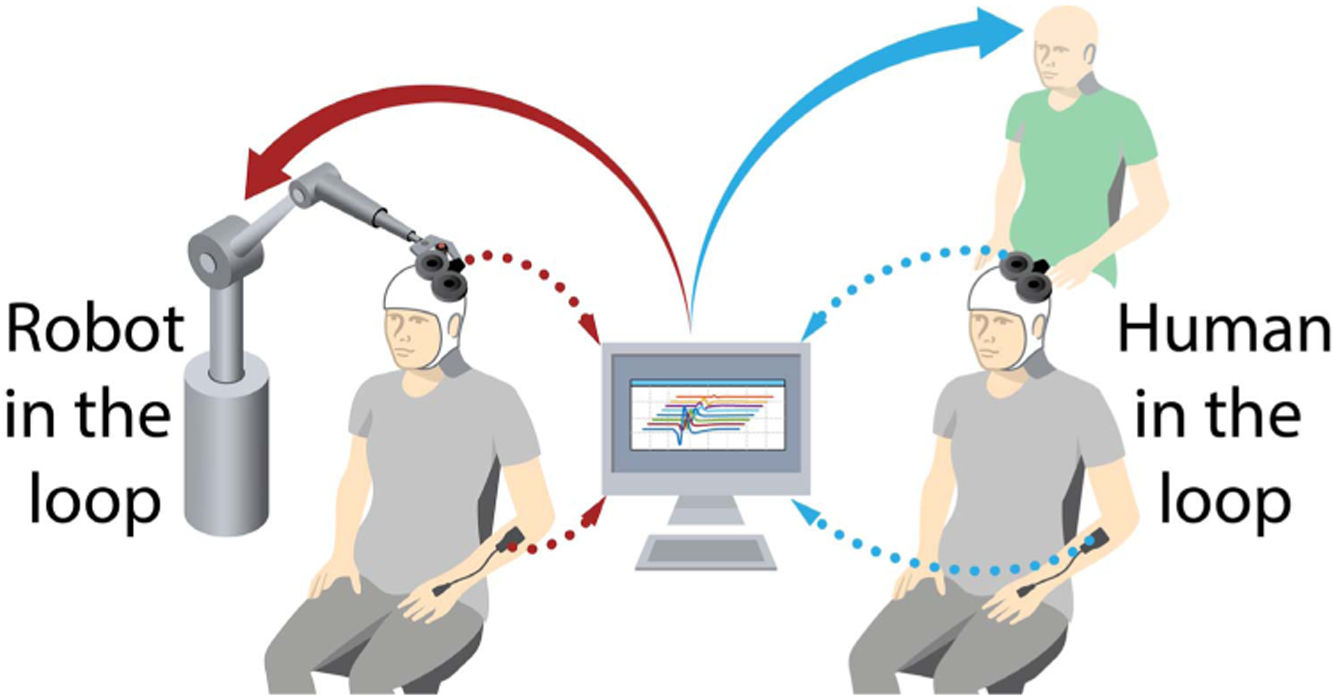
Illustration of a framework where operator expertise (right) for selection of stimuli in the user-guided mapping approach is replaced with machine learning and robotics(left). Here, a machine-learning-driven robot is shown to illustrate autonomous mapping.

The USRG approach could be categorized as a form of implicit active learning (a field of machine learning) [16], [17], since the coil operator utilizes an implicit objective function built on experience and expertise to select stimulation loci. The goal of this work is to replace the TMS operator’s expertise with an automated algorithm based on *statistical* active learning. This kind of automated approach will not only result in more objective, time efficient, and repeatable sampling; it will also be particularly useful for TMS mapping that utilizes new advances in robotic coil positioning [18], as it allows the development of fully autonomous, efficient mapping. Figure 1 illustrates such a framework, in which TMS operator expertise in the USRG mapping approach can be replaced with robotic coil control guided by active learning.

The topic of active sample selection has been investigated in many fields including applications to optimal sensor placement [19]-[21], active GP sampling [22], and weather forecasting [23]. Ideally, given an optimality criterion, the goal is to select a limited number of samples that “best” represents the phenomenon of interest (e.g., here the field of MEP responses). One approach to this problem statement is to employ greedy on-line strategies where at each step the algorithm searches for the next sample location that leads to the highest one-step gain in the chosen optimality criterion. One statistical approach that can be used to model a spatial phenomena is Gaussian Processes (GPs), an approach for probabilistic regression in reproducing kernel Hilbert spaces that allows us to explicitly model uncertainty about estimated function values. GPs provide a well-defined yet flexible stochastic framework which can be exploited to compute and optimize the desired criteria [24]. Crucially, GPs provide both a spatial interpolation and an estimate of model uncertainty as a function of space [20]. These two quantities are associated with the Gaussian representation of the spatial field and allow the derivation of analytical expressions for conditional entropy that are typically needed for information theoretic criteria. In recent studies, several optimal point selection strategies have been presented in conjunction with GPs [19], [20], [24]. In these works, the authors considered both entropy-based and mutual information (MI)-based criteria, providing greedy, near-optimal strategies. These strategies succeeded in reducing the GP’s spatial uncertainty, because the variances provided by the GP depend exclusively on the *location* of points, while completely neglecting available information regarding the field’s *amplitude* variations. This implies that even when the changes in the field’s amplitude is confined to a specific subregion, these methodologies will lead to a relatively even sampling of the entire space (as determined in detail by the selection of the GP kernel, which governs its spatial covariance structure) [16]. In problems such as TMS mapping, where the Region of Interest (ROI) is completely or largely confined to a subregion of the space, this tendency towards evenly-spaced sampling reduces resolution in the region where the activity is concentrated. A good criterion for automated TMS mapping should balance exploration and exploitation to discover the ROI and concentrate most of the samples in that region.

We previously reported an initial formulation of an alternative GP-based information-theoretic strategy relying on amplitude uncertainty instead of spatial uncertainty [16]. This was accomplished by modeling the mean of the GP as a GP itself, and considering information-based criteria which led to selecting samples that reduced uncertainty across MEP amplitude values. One drawback of this formulation was that it neglects spatial uncertainty, causing it to get stuck in areas with high MEP amplitudes while ignoring unexplored regions of the space—in effect favoring exploitation over exploration. Inspiration for alternative approaches can be found in the use of active sampling in other domains. Specifically, Gunter *et al.* proposed an active sampling strategy as a tool for Bayesian Quadrature integration which leveraged the fact that the GP regression is being used to approximate likelihood functions, and, thus, must be non-negative [25]. To match this observation, the authors considered GPs with a square-root warping function (see (5)) which has several desirable properties, including guaranteed non-negativity for the observation model, and a covariance function which naturally balances exploration and exploitation. Another alternative sampling algorithm might be considered where the balance between exploration and exploitation depends exclusively on the shape of the GP mean modeled as a probability density function (pdf). For instance, Imbiriba *et al.* proposed a GP-based sampling algorithm developed in the context of particle filtering, where samples were drawn from a GP mean function that was normalized in order to make it a viable pdf [26]. This strategy presents some features that fit well with TMS mapping. First, it naturally concentrates most samples in the ROI. Second, it also leads to random exploration of the space in which the compromise with exploitation will be automatically controlled by the concentration of the field’s amplitude.

In this paper we investigate the GP-based active learning strategies discussed above for the automated TMS mapping problem. We report on two studies. First, we compared the performance of experimental (traditional) sampling approaches (regular grid sampling (GRID), random sampling (RAND), and USRG) acquired from 5 individuals, and second, we compared the performance of computational GP-based strategies for the reconstruction of the experimentally acquired maps with respect to the time efficiency and accuracy of the maps. We hypothesized that a computational GP-based strategy can be used to achieve at least similar accuracy to an expert user-guided sampling approach with equal or greater efficiency.

## II. Gaussian Process for TMS Mapping

GPs can be viewed as interpolators capable of providing Gaussian distributions for every point in the space. This stochastic formulation provides important features that are relevant here, in particular that GPs provide model uncertainty measures as a function of location. This quantified model uncertainty can be used to aid in trading off between exploration and exploitation. In addition, having a probabilistic model enables the use of methods based on information theoretic criteria. The GP formulation defines stochastic models directly in the functional space by assuming Gaussian priors for functions ψ∈H, ψ∣x∼N(0,κ(x,x)), where H is a functional Hilbert space, *κ* is a covariance function or kernel, and $x\in X\subset R^{d}$ are the inputs of the system of interest. We assume that we observe an “output” ***y*** corresponding to every ***x***. Then, given a set of *N* input-output pairs $\{x_{k},y_{k}{\}}_{k\in A}$, where y∈R and A is an index set such that ∣A∣=N, GP regression aims at providing a predictive distribution for a new sample ***x***_*ℓ*_ conditioned on the training set. This can be accomplished by assuming a Gaussian model relating inputs and outputs:

(1) $$y_{k}=\psi (x_{k})+\eta_{k}$$

where $\eta ∼N(0,\sigma_{\eta }^{2})$. The predictive distribution [27] for a new input ***x***_*ℓ*_ can be obtained as:

(2) $$\psi |y_{A},X_{A},x_{\ell }∼N({\hat{\psi }}_{\ell |A},s_{\ell |A}^{2})$$

with ${\hat{\psi }}_{\ell |A}=\kappa_{A\ell }^{T}{\left(K_{AA}+\sigma_{\eta }^{2}I\right)}^{-1}y_{A}$, and $s_{\ell |A}^{2}=\kappa_{\ell \ell }-\kappa_{A\ell }^{T}{\left(K_{AA}+\sigma_{\eta }^{2}I\right)}^{-1}\kappa_{A\ell }$, where $\kappa_{A\ell }=\kappa (X_{A},x_{\ell })$, $\kappa_{\ell \ell }=\kappa (x_{\ell },x_{\ell })$, and $X_{A}\;=\;[x_{A_{1}},\ldots ,x_{A_{N}}{]}^{T}$. For a detailed derivation see [27]. The Bayesian framework also provides strategies to estimate free parameters, such as the kernel parameters ***θ*** and the noise power $\sigma_{\eta }^{2}$. We consider the exponentiated-quadratic kernel with $\theta \;\in \;R^{2}$ containing the scaling and kernel bandwidth parameters, with maximal likelihood kernel parameter selection [27] that minimizes (3):

(3) $$-2\;\text{ln}\;f_{Y}(y_{A}|X_{A},\sigma_{\eta }^{2},\theta )=y_{A}^{T}K_{AA}^{-1}y_{A}+\text{ln}\;|(2\pi )K_{AA}|$$

with respect to ($\sigma_{\eta }^{2}$, ***θ***).

GP regression has been successfully used in a wide variety of fields including regression and classification [27], detection [28], [29], unmixing [30], and Bayesian optimization [31]. One limitation of GP is related to the Gaussian assumption on the posterior distribution, which makes standard GP models unfit or inaccurate when the observation distribution is non-Gaussian. This is the case for TMS mapping where measured MEP amplitudes are inherently non-negative quantities. To circumvent this issue for our setting, a mapping function can be applied to Gaussian random variables to enforce non-negativity, resulting in non-Gaussian random variables. These procedures with non-linear mappings are known as Warped GPs (WGPs) [25], [32], which we describe in detail next.

### A. Warped Gaussian Process

To guarantee non-negativity of the functions being modeled and at the same time exploit the GP framework, a common strategy is to consider a warping function $g\;:Y\subset R_{+}\to Z\subset R$, *Y* ↦ *Z*, where *Y* is a non-negative random variable. Let *Z* and *Y* be two multivariate random variables, whose realizations are ***z*** and ***y***, related through a mapping function *g* as *Z* = *g*(*Y*). The densities of *Z* and *Y* are related [33] according to (4).

(4) $$f_{Y}(y)=f_{Z}(g(y))|J_{Y}^{-1}|$$

where $J_{Y}=\frac{\partial (z_{1},\ldots ,z_{N})}{\partial (y_{1},\ldots ,y_{N})}$ is the Jacobian matrix. This allows exchanging learning in the original space for learning in the latent warped space Z. Thus, using (4), fitting a GP in the latent space reduces to maximizing the GP’s log marginal likelihood function [32]. Different warping functions have been considered in the literature to cope with the requirements of different applications [25], [32].

In the context of TMS mapping, warping functions must guarantee non-negativity of the estimated field without imposing hard upward limits. Thus, in this work, we selected the square-root warping function given by:

(5) $$z=g(y)=\sqrt{2(y)},\;\;y=g^{-1}(z)=\frac{1}{2}z^{2}$$

## III. Active GP Sample Selection

A general problem formulation for active sample selection consists of selecting a set of input-output pairs $A≔\{x_{\ell },y_{\ell }{\}}_{\ell =1}^{n_{A}}$, with cardinality $|A|=n_{A}$, such that the loci $x_{\ell }\in R$ where the measurements ***y***_*ℓ*_ take place are selected following some optimality criterion. In many scenarios where the ROI R is discretized, i.e., ℓ∈V with V an index set of all discretized locations in R, the problem can be stated as optimally selecting the subset A such that ℓ∈V and $n_{A}\;\ll \;|V|=n_{V}$. As mentioned, ideally, given an optimal criterion, the goal is to select $n_{A}$ samples that “best” represent the ROI. Next, we discuss the main types of information theoretic-based active sample selection methods that will be analyzed in this paper.

### A. Standard Information Theoretic-Based Sampling

Information based criteria generally focus on selecting samples that lead to the highest decrease in the uncertainty (entropy) or that are the most informative about unsensed locations (MI) [20]. Since global approaches based on such criteria lead to NP-complete problems [20], [34], greedy iterative strategies are often employed such that, at each iteration, the algorithm will select the locus that leads to the maximum decrease in entropy or maximum increase in MI. Thus, considering V as defined, and letting A denote the index set of the loci already selected at some arbitrary iteration, then active selection selects the index *ℓ* that leads to the maximum gain according to the selected criteria at each iteration, that is:

(6) $$\ell^{*}=\underset{\ell \in V\setminus A}{\text{arg}\;\max}\delta_{\ell }^{\text{Criterion}}$$

where $\delta_{\ell }^{\text{Criterion}}$ is the adopted criterion quantity and V∖A is the set of all indexes in V that are not in A.

When considering entropy, the goal of GP-based greedy algorithms is to, iteratively, select a locus that results in the largest conditional entropy given by (7) among all of available measurement locations ℓ∈V∖A, conditioned on measured loci $y_{A}$:

(7) $$H(\psi_{\ell }|y_{A})=-\int p(\psi_{\ell },y_{A})\;\log\;p(\psi_{\ell }|y_{A})d\psi_{\ell }dy_{A}$$

The conditional entropy for Gaussian random variables [35] is well known and given by (8).

(8) $$H(\psi_{\ell }|y_{A})=\frac{1}{2}\;\log\left(2\pi s_{\ell |A}^{2}\right)+\frac{1}{2}$$

where $s_{\ell |A}^{2}$ is the GP variance, leading to a simple and effective selection strategy. Since log(*ζ*) is monotonically increasing for $\zeta \in R_{+}$, problem (6) can be solved for GP entropy by finding the sample index ℓ∈V∖A, that maximizes the quantity:

(9) $$\delta_{\ell }^{\text{Entropy}}=s_{\ell |A}^{2}=\kappa_{\ell \ell }-\kappa_{A\ell }^{T}K_{AA}^{-1}\kappa_{A\ell }.$$

A known issue with the entropy criterion is that it has the tendency to select loci along the edges of the sample space. This can be explained by the fact that the entropy grid approach aims at selecting the *ψ_ℓ_* with the largest variance $s_{\ell |A}^{2}$, and these uncertainties are known to be larger on the edges of the sampling space [36].

An alternative approach is based on the MI of random variables within the set A and in V∖A. This strategy leads to an optimization criterion that searches for the subset of locations that most significantly reduces the uncertainty about the estimates in the rest of the space [20]. Problem (6) can be solved for GP MI by finding the sample index ℓ∈V∖A that maximizes the quantity (for more detail see [20]):

(10) $$\delta_{\ell }^{\text{MI}}=\frac{\kappa_{\ell \ell }-\kappa_{A\ell }^{T}K_{AA}^{-1}\kappa_{A\ell }}{\kappa_{\ell \ell }-\kappa_{\bar{A}\ell }^{T}K_{\bar{A}\bar{A}}^{-1}\kappa_{\bar{A}\ell }}$$

where $\bar{A}=V\setminus (A\cup \ell )$, and $K_{QQ}$ is the kernel matrix for all loci ***x***_*ℓ*_ with ℓ∈Q.

MI-based strategies, different from entropy, tend to find loci that are most informative about the unstimulated locations, thus avoiding sampling the edges [20]. One drawback of MI active sampling strategies is the large computational complexity of the resulting greedy algorithms, since at each iteration the computation of (10) requires matrix inverses whose sizes are proportional to all points available ($|\bar{A}|$).

Previously, we showed that although MI can lead to faster initial convergence because it does not initially focus on sampling the edges, for the TMS mapping problem, the two methods perform very similarly in practice as the number of samples increases, providing loci that are almost uniformly placed on the scalp over the motor cortex [16]. For this reason, we only consider entropy-based strategies in this paper.

### B. Amplitude-Based Entropy Sampling

To overcome the limitation that naive entropy-based active sampling strategies tend to lead to uniformly distributing sampling loci, in our previous study we proposed an alternative approach that takes into account the variations of the function *ψ*, leading to a sampling strategy that seemed intuitively to more closely match the USRG strategy [16]. We used the GP estimate itself (i.e., the GP mean) $\hat{\psi }$ to construct an iterative strategy where GP estimation and sample selection were performed sequentially. Thus, given an index set A with respective sample pairs {$X_{A}$, $y_{A}$} the GP estimate ${\hat{\psi }}_{p}$ for all p∈V can be obtained by taking the mean of (2). Assuming a zero-mean Gaussian prior for ${\hat{\psi }}_{p}$ with covariance *κ*(·, ·), we have

(11) $${\hat{\psi }}_{p}∼N(0,{\tilde{\kappa }}_{pp})$$

with ${\tilde{\kappa }}_{pp}≜\kappa ({\hat{\psi }}_{p},{\hat{\psi }}_{p})$.

The MEPs at selected indices ${\hat{\psi }}_{A}$ are distributed

(12) $${\hat{\psi }}_{A}∼N(0,{\tilde{K}}_{AA})$$

with ${\tilde{K}}_{AA}≜\kappa ({\hat{\psi }}_{A},{\hat{\psi }}_{A})$. Now, if we consider a sample ${\hat{\psi }}_{\ell }$, ℓ∈V∖A, the joint distribution of ${\hat{\psi }}_{\ell }$ and ${\hat{\psi }}_{A}$ is given by

(13) $$\left(\begin{array}{c} {\hat{\psi }}_{A}\\ {\hat{\psi }}_{\ell } \end{array}\right)∼N\left(0,\left(\begin{array}{cc} {\tilde{K}}_{AA} & {\tilde{\kappa }}_{A\ell }\\ {\tilde{\kappa }}_{A\ell }^{T} & {\tilde{\kappa }}_{\ell \ell } \end{array}\right)\right),$$

with ${\tilde{\kappa }}_{A\ell }≜[\kappa ({\hat{\psi }}_{A_{1}},{\hat{\psi }}_{\ell }),\ldots ,\kappa ({\hat{\psi }}_{A_{n_{A}}},{\hat{\psi }}_{\ell }){]}^{T}$. Using the identity in [27] we have ${\hat{\psi }}_{\ell }|{\hat{\psi }}_{A}∼N\left(\mu_{{\hat{\psi }}_{\ell |A}},\sigma_{{\hat{\psi }}_{\ell |A}}^{2}\right)$ with

(14) $$\mu_{{\hat{\psi }}_{\ell |A}}={\tilde{\kappa }}_{A\ell }^{T}{\tilde{K}}_{AA}^{-1}\psi_{A},\;\;\sigma_{{\hat{\psi }}_{\ell |A}}^{2}={\tilde{\kappa }}_{\ell \ell }-{\tilde{\kappa }}_{A\ell }^{T}{\tilde{K}}_{AA}^{-1}{\tilde{\kappa }}_{A\ell }.$$

Adopting the entropy-based criterion for sample selection with this amplitude-based approach leads to a sample choice criterion: $\delta_{\ell }^{\text{Criterion}}≜\delta_{\psi ,\ell }^{\text{Entropy}}$ as:

(15) $$\delta_{\psi ,\ell }^{\text{Entropy}}=\sigma_{{\hat{\psi }}_{\ell |A}}^{2}={\tilde{\kappa }}_{\ell \ell }-{\tilde{\kappa }}_{A\ell }^{T}{\tilde{K}}_{AA}^{-1}{\tilde{\kappa }}_{A\ell }.$$

### C. Entropy With Warped GPs Sampling

To ensure non-negativity, Warped GPs can be leveraged as discussed in Section II-A where the invertible mapping g:Y→Z between the MEP space Y and a latent space Z was presented. Thus, equation (5) leads to $Z=g(Y)=\sqrt{2Y}$. Now, assuming a Gaussian prior on Z:Z∣x∼N(0,κ(x,x)) the predictive distribution for a new location ***x***_*ℓ*_ can be written as: $Z|z_{A}$, $X_{A}$, $x_{\ell }∼N\left(\mu_{z_{\ell |A}},\sigma_{z_{\ell |A}}^{2}\right)$ with

(16) $$\mu_{z_{\ell |A}}=\kappa_{A\ell }^{T}K_{AA}^{-1}z_{A},\;\;\sigma_{z_{\ell |A}}^{2}=\kappa_{\ell \ell }-\kappa_{A\ell }^{T}K_{AA}^{-1}\kappa_{A\ell }.$$

Since linear transformations of GPs are also GPs, considering a local linearization of the inverse mapping $Y=\frac{1}{2}Z^{2}$ leads to a GP over *Y* given a GP defined over *Z* [25]. Specifically, considering the first order Taylor expansion of *g*^−1^, we have $y≃g^{-1}(z_{0})+\frac{\partial g^{-1}(z)}{\partial z}{|}_{z=z_{0}}(z-z_{0})$.

**Table:** 

Algorithm 1 Active GP Entropy-Based Sampling Algorithm
Input:XA0,yA0,V,XV,N,methodOutput:A1Obtain estimated fieldψ^Vby taking the mean of (2) (forGPE and GPEμ)or(18)(for WGPE);2Setn=n0andA=A0;3whilen≤Ndo4∣forℓ∈V∖Ado∣Computeδψ,ℓusing (9) or (15) or (20) based onmethod;∣endℓ∗=argmaxℓδψ^,ℓ;Get MEP sampleyℓ∗at locationxℓ∗;A=A∪{ℓ∗};V=V−{ℓ∗};Obtain estimated fieldψ^Vby taking the mean of (2)or(18);Increment n;∣13end14returnA;

One interesting characteristic of the mapping *g* is that the derivative $\frac{\partial g^{-1}(z)}{\partial z}=z$. Thus, choosing $z_{0}=\mu_{z_{\ell |A}}$ leads to:

(17) $$y≃\frac{1}{2}\mu_{z_{\ell |A}}^{2}+\mu_{z_{\ell |A}}\;(z-\mu_{z_{\ell |A}})$$

and using the approximation $z≃\mu_{z_{\ell |A}}$, we have:

(18) $$Y|y_{A},X_{A},x_{\ell }∼N\left(\mu_{y_{\ell |A}},\sigma_{y_{\ell |A}}^{2}\right)$$

with

(19) $$\mu_{y_{\ell |A}}=\frac{1}{2}\mu_{z_{\ell |A}}^{2},\;\;\sigma_{y_{\ell |A}}^{2}=\mu_{z_{\ell |A}}^{2}\sigma_{z_{\ell |A}}^{2}.$$

Consequently, the entropy criterion leads to solving problem (6) using (19):

(20) $$\delta_{Z,\ell }^{WGP}=\sigma_{y_{\ell |A}}^{2}=\mu_{z_{\ell |A}}^{2}\sigma_{z_{\ell |A}}^{2}.$$

One important consequence of the selected mapping *g* and the linearization of its inverse is the fact that the variance in (19) and, consequently, the quantity (20), are functions of the GP’s variance and mean. This implies that this approach balances exploration and exploitation naturally by selecting loci where the model has high uncertainty (large $\sigma_{z_{\ell |A}}^{2}$) and high amplitude (large $\mu_{z_{\ell |A}}$).

### D. Entropy Based Sampling Algorithm

Algorithm 1 presents the proposed active GP entropy-based sampling strategies. It is designed to work with a set of initial loci $X_{A_{0}}$ and their respective MEPs $y_{A_{0}}$, a set of selectable indices V with associated loci $X_{V}$, the final cardinality *N*, and finally the chosen entropy-based method. In line 7, a new index ℓ* is selected based on a chosen method given by (9), or (15), or (20). We refer to these methods as: standard GP entropy (**GPE**), Amplitude-based GP entropy (**GPE**_*μ*_), and Warped GP entropy (**WGPE**) respectively. The algorithm follows an iterative sequence interchanging between computing *δ*_*ψ,ℓ*_ for all ℓ∈V∖A (lines 4–6), finding the optimal index (line 7), updating the sets and MEP field estimation (lines 9–11). When the desired cardinality (N) is achieved the algorithm returns the set of selected (suggested) stimuli loci and the corresponding estimated MEPs.

To have a baseline for the purpose of comparison we also implement a naive uniform grid sampling (**UGRID**) strategy by considering a uniform grid of N points over the space at each iteration.

## IV. Adaptive GP Random Sampling

An entirely different possible sampling strategy for TMS mapping would consist of obtaining sample locations by randomly selecting locations according to an appropriate distribution *p*(***x***) defined over X. A naive approach would be to uniformly sample over the entire motor cortex. A more principled approach, however, would exploit the knowledge provided by iteratively observing the MEP values acquired by previous samples to update an estimate of this distribution and, thus, gradually concentrate future samples in the area where previous MEPs had larger amplitudes (namely, ROI).

Imbiriba *et al.* proposed a GP-based sampling methodology in the context of particle filtering where, at each iteration, the GP’s mean function was used to approximate the posterior distributions of particle filters and in the resampling process [26]. To adopt this strategy to the problem at hand we need to choose an appropriate model for the probability density function (PDF) to update. We note that a function *p*(***x***) is a valid PDF if it obeys two basic properties: 1) non-negativity and 2) it integrates to one. Non-negativity can be achieved here by leveraging Warped GPs as discussed in Section II-A while the integration-to-one requirement can be achieved by properly normalizing the WGP mean function. That is, if we let *μ*(***x***) be the mean function of a non-negative WGP, then

(21) $$\hat{p}(x)=\mu (x)\;∕\;\int \mu (x^{\prime })dx^{\prime }$$

is a valid PDF.

Algorithm 2 presents the Gaussian Process Random Sampling (**GPRS**) algorithm. It receives as inputs an initial set of loci and measurements {***X***_0_, ***y***_0_} with cardinality *n*_0_, the final number of points *N* and the initial distribution ${\hat{p}}_{n_{0}}(x)$. Then, an iterative procedure is performed by randomly sampling a new location $x_{\ell }∼{\hat{p}}_{\ell }(x)$, measuring a new MEP *y*_*ℓ*_ at ***x***_*ℓ*_ and updating the distribution by using all available pairs ***X**, **y***. The update performed in line 6 involves updating the WGP and normalizing it according to (21). The sampling procedure at line 3 can be performed using any strategy aimed at sampling arbitrary distributions such as Accept-Reject, Importance Sampling, etc [37]. In this work, we opted for the Accept-Reject method due to its simplicity. In order to have a baseline for purpose of comparison we also implement a naive random sampling strategy by considering *p*(***x***) as a fixed uniform distribution in the area of interest (e.g. over the motor cortex). We refer to this approach as Uniform Random Sampling (**URAND**).

**Table:** 

Algorithm 2 Adaptive GP Random Sampling Algorithm
Input:X0,y0,n0,nA,p^n0(x)Output:X,y1X=X0;2forℓ=n0…,Ndo3∣Samplexℓfromp^ℓ(x);Get MEPyℓat locixℓ;MakeX=[XT,xℓT]Tandy=[yT,yℓ]T;Updatep^ℓ(x)using the pair{X,y};∣7end8returnX,y;

An interesting difference between the main two sampling strategies proposed in this paper (Active GP Entropy-based and Adaptive GP Random Sampling) is that the latter does not require a grid and incurs a much lower computational cost, since it does not need to evaluate the quantity $\delta_{\ell }^{\text{Criterion}}$ for all points in a grid. On the other hand, its results are, obviously, variable from one run to the next due to the random draws from the density.

## V. Experimental Setup

In this section we present our experimental setup. First, we discuss the non-automatic TMS mapping strategies used to collect real data. Then, we discuss how we used this collected data to build a realistic data set with accessible “ground truth”. Finally, we present the metrics used for assessing the performance of the different methodologies.

### A. TMS Mapping Procedure

All protocols were conducted in conformance with the Declaration of Helsinki and were approved by the Institutional Review Board of Rutgers Biomedical Health Sciences, Newark, NJ. Five healthy, right handed subjects (3 males & 2 females) aged 22 to 40 participated following written informed consent. Participants were free from neurological and musculoskeletal deficits, and systemic disease. Exclusion criteria included contraindications for TMS, including metallic or electronic implants in the head, pregnancy, and history of epilepsy. Subjects were seated with their arm, hand, and fingers comfortably secured in a brace to limit motion. Surface electromyographic activity (EMG, Delsys Trigno, 1kHz) was recorded from the first dorsal interosseous (FDI) of the right hand. To record the location of TMS (Magstim, Rapid^2^) stimuli, each subject’s head was coregistered to a canonical high-resolution anatomical MRI for frameless neuronavigation (Advanced Neuro Technology). The TMS coil (Magstim, 70 mm double coil) was held tangential to the scalp, with the handle posterior 45° off the sagittal plane [7], [38], [39]. MEPs were quantified as the peak-to-peak (pp) amplitude of the FDI EMG signal in a window from 10 to 40 ms following the TMS pulse. The cortical hotspot was found by sampling the hemisphere until the locus with the largest MEP was located [8], [40]. Resting motor threshold (RMT) was determined at this location as the minimum intensity required to elicit MEPs > 50 *μ*v in the FDI muscle on 3 of 6 consecutive trials [38], [41]. All TMS measures were taken at rest and background EMG was monitored to ensure that muscles remained relaxed. All mapping was performed with the stimulation intensity set to 110% of RMT [42]. Each mapping included 294 stimulations, delivered at an interstimulus interval of 4.2 ±.25 seconds, necessitating ~20 minutes to complete. Mapping of the right FDI representation on the left sensorimotor cortex using a GRID, RAND, or USRG-based spatial sampling approach were performed in a single session with 5-10 minute breaks between mappings. Order of the mapping was randomized between subjects. Details of each method are as follows: 1)GRID: A 7 × 7 grid (49 points, 36 cm^2^) with 1 cm spacing between grid sites was centered at the FDI hotspot in the tangential plane to the scalp. To place the grid on the scalp, head model data was exported from the neuronavigation software into Matlab (Mathworks). Grid points were translated inward towards the scalp along a vector intersecting the center point of the standard head model in order to project the grid directly onto the scalp. The grid was then imported into the neuronavigation software and superimposed virtually on the head model. Each of these grid sites was stimulated 6 times. 2) RAND: The random sampling included the 4 corners of the grid, the FDI hotspot, and 289 uniform randomly distributed sites (294 total) over the same 6 × 6 cm area. These sites were again projected onto the scalp using the same procedure described for GRID. One stimulus was delivered to each site in the random set. 3) USRG: Only the 4 corners of the grid and the site signifying the FDI hotspot were projected onto the scalp. At the beginning of the experiment, stimulation was applied to each of these sites once (5 stimulations). The remaining 289 stimulations were chosen at the discretion of the TMS operator based on real-time feedback of MEP amplitude. The intention of the operator was to maximize the information obtained per stimulus by increasing the density of points in what they judged at the time to be excitable and border regions while placing few points in what they judged to be null-response areas [12], [13].

### B. TMS Dataset for Computational Experiments

To test the computational methods, we created an accessible “ground truth” (GT) function from the *combination* of three experimental maps (GRID, RAND, USRG). We then used this function as a surrogate to determine the MEP amplitude obtained from any arbitrary sampling location chosen by an algorithm. This is crucial for our computational methods that use the real-time feedback of MEP amplitude to select future loci. To do so, two interpolation steps were used. In the first step cubic interpolation was used to interpolate all available points to a 3.75 mm resolution grid as has been previously described by our group and others [4], [38], [43]. We then used this interpolated data as a training data for WGP model (over GP, to ensures non-negativity), calculated the posterior, and computed the predictive posterior distribution (see section II) on any point of interest ***x***. Thus, for any of the methods tested, once a sampling location is determined, its associated MEP amplitude can be obtained from this predictive distribution.

For the Active GP Entropy-based Sampling methods that need a set of selectable loci (for greedy approach), we used a grid with 2 mm resolution in 2D space. This resolution seemed good based on real world TMS mapping resolution and map liability. We discuss more about this resolution in the section VII. For Adaptive Random Sampling methods, as stated before, we do not require a grid and methods should choose any arbitrary location in the space.

### C. Assessment of Accuracy of the Sampling Methods

To assess the accuracy of the methods, we created a testing data including a dense grid of 0.5 mm resolution loci in 2D space and their associated MEP amplitudes (GT values). Reconstruction error was defined as the normalized mean squared error (NMSE) between GT MEP values and predicted MEP values from selected loci by each method for testing data. Specifically, we defined NMSE as: $\text{NMSE}(\psi ,\hat{\psi })=(\|\psi -\hat{\psi }{\|}_{2}^{2})∕(\|\psi {\|}_{2}^{2})$ where ***ψ*** is a vector containing the GT MEPs and $\hat{\psi }$ is a corresponding vector of MEPs obtained using the sample points chosen by a particular method. In addition to NMSE, we computed several common map features as outcome measures: map volume, map area, and center of gravity in both the rostral-caudal plane (denoted COGx1) and in the medial-lateral plane (COGx2). Map volume and map area were calculated using double trapezoidal integration of the interpolated maps [44]. COGx1 and COGx2 were computed using standard equations [42].

## VI. Results

**Fig. 2.**
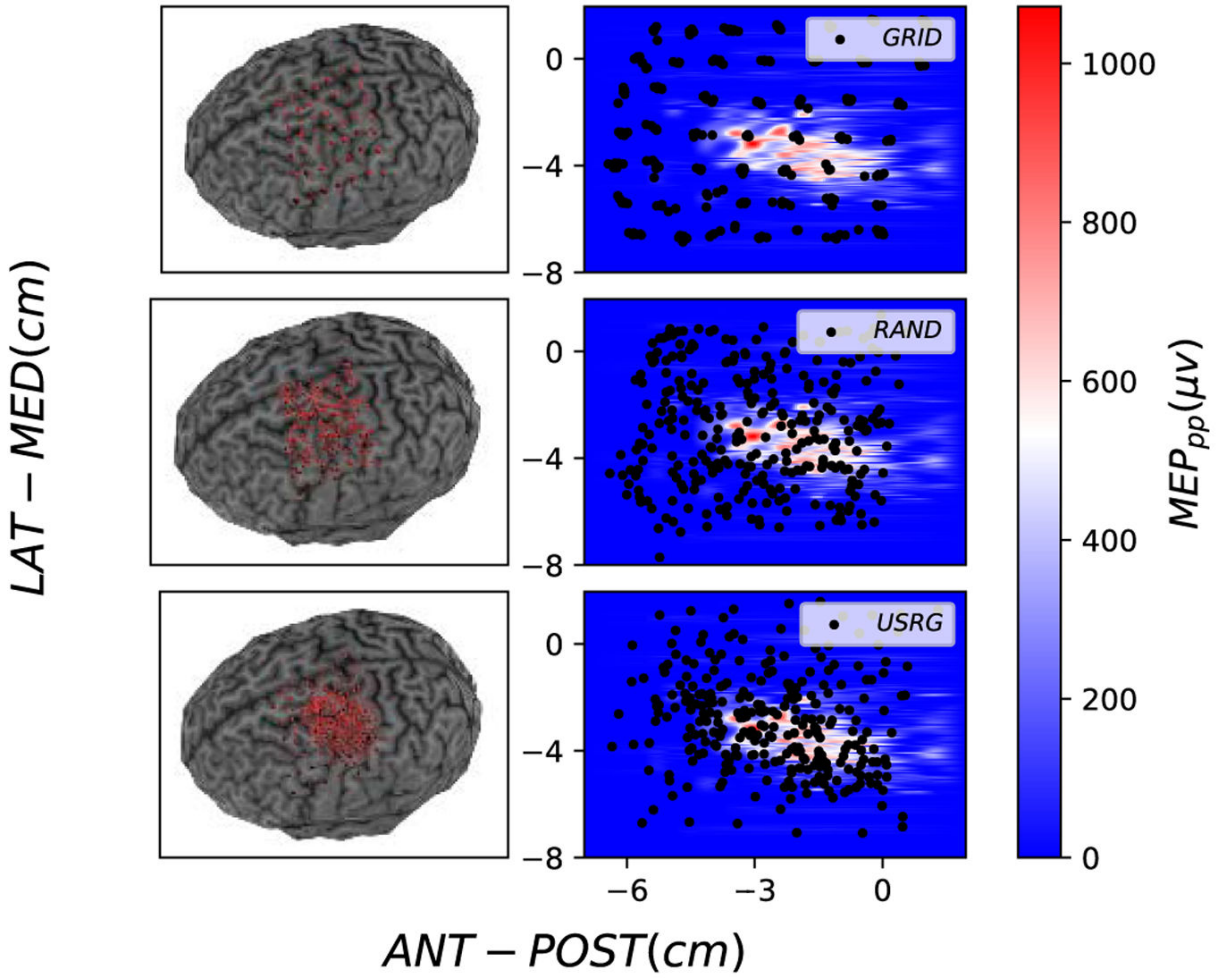
Stimulation loci (Left: red, right: black circles) for GRID (top), RAND (middle) and USRG (bottom) mappings shown on the **Left:** 3D curvilinear canonical brain used for neuronavigation and **Right:** Heat maps indicating interpolated 2D map representation of MEP amplitude in *μ*V for subject 3. The color bar represents the MEP_*pp*_ and axes display the spatial coordinate system used by the neuronavigation software which places the origin at the intersection of the fiducial markers used for co-registration (roughly at the center of the brain).

Mapping procedures were well tolerated by all 5 subjects and no adverse effects of the stimulation were reported. Stimulation loci selected by each experimental method (GRID, RAND, USRG) are shown for subject 3 in Figure 2. Map features (area, volume, COGx1, and COGx2) determined for the maximum number of sampled loci are shown for the GT and each collected map in the left-most columns (Experimental Methods) in Table I. Group Mean (GM), defined as the average across subjects, MapArea and MapVol were observed to be smaller for GRID sampling, than for RAND or USRG sampling (see last row of Table I). To illustrate how the error in the acquired experimental maps depends on the method as more samples are acquired, the evolution of NMSE between GT and each experimental map across the duration of mapping is shown for all subjects in the first row of Figure 4. Lower GM NMSE was observed for USRG and RAND when compared with the GRID approach (GM NMSE: 0.36±0.11, 0.68±0.17, 0.99±0.00 respectively) after 49 simulations. The GRID approach resulted in less reduction of NMSE with added stimuli compared to RAND or USRG. USRG outperformed RAND (GM NMSE: 0.33±0.10, 0.60±0.18 respectively) following 100 stimuli for all 5 subjects, and for some subjects increased performance was seen with fewer stimuli. RAND and USRG became more similar in terms of NMSE with added stimuli in all subjects; however USRG provided a better fit to the GT in every case.

**Fig. 3.**
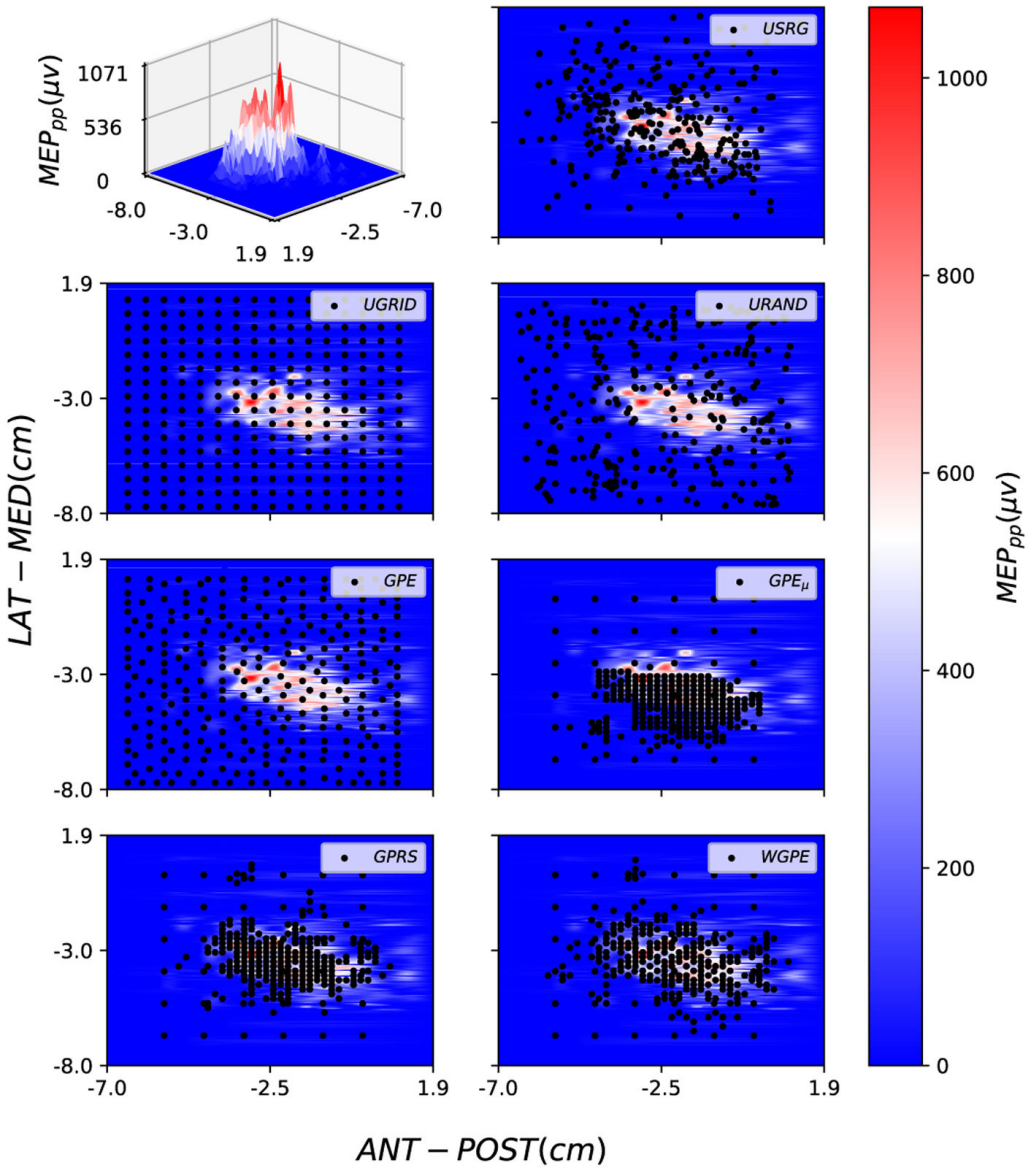
Using Warped GP model, a surface is fitted on the real TMS data to create a representation of MEP map for subject 3. This map can be viewed in either a 3D (top left) or 2D (others) map. The color bar represents the MEP_*pp*_ and Black circles indicate the selected stimulation loci by each method (specified in top right of each subplot).

**Fig. 4.**
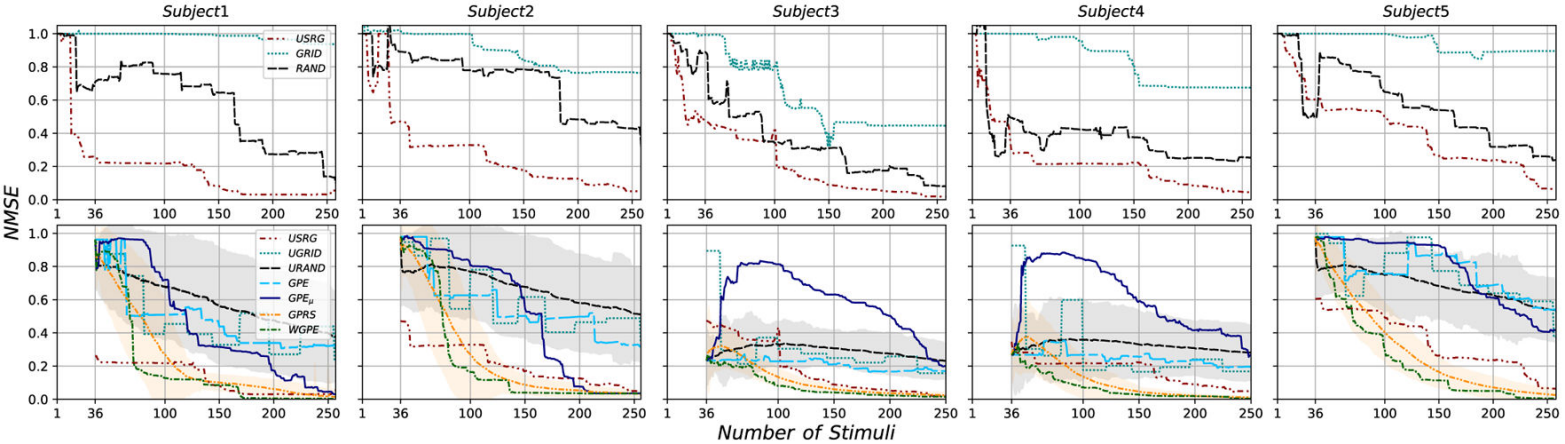
NMSE between GT and predicted MEP values for testing data as the number of stimuli are added by each experimental method (**First row**) and computational method (**Second row**) for all subjects. NMSE mean (line) and Standard deviations (transparent shade) are presented for URAND and GPRS methods for which Monte Carlo procedures were used to understand randomness in stimuli selection.

For all computational methods, an initial uniform grid set $A_{0}$ with cardinality $|A_{0}|=36$ was selected. We selected a total of 256 stimuli with each of the computational methods. Figure 3 presents details of the loci of the stimuli selected by each method for subject 3. To evaluate the extent to which each algorithm samples the excitable region of the cortex the percentage of sampled stimuli with a MEPs greater than 50*μ*V (the threshold used to determine RMT, see Sec. V-A) were determined. Averaged across participants, the methods that placed a high percentage of new stimuli in the ROI (excitable region) were GPE_*μ*_ (67±16%), GPRS (79±12%), and WGPE (78±13%), while UGRID (12±4%), URAND (14±3%), and GPE (12±3%) spread stimuli more uniformly over the motor cortex. This resulted in the GPE_*μ*_, GPRS, and WGPE approaches being qualitatively more similar to the USRG approach (40±5% stimuli in ROI) both in terms of behavior as a function of number of stimuli as well as the final sample map appearance and progression. Figure 4 (second row) shows the evolution of NMSE between the GT and the maps from each of the computational methods as a function of increasing number of stimuli. Monte Carlo simulations were performed for methods with randomness (URAND and GPRS) and for those methods we show the mean (solid color) and standard deviation (STD) (transparent shade) in the figure. Lower GM NMSE after 100 simulations was observed for USRG, GPRS, and WGPE (0.33±0.10, 0.24±0.10, 0.14±0.07 respectively) when compared with GPE_*μ*_, GPE, URAND, and UGRID (0.82±0.07, 0.48±0.20, 0.58±0.19, 0.52±0.27 respectively). The best performing methods, WGPE and GPRS, achieved lower NMSE than the operator-dependent USRG method at GM number of stimuli: 65.25±13.93 and 90.25±10.33 respectively. In 4 of 5 participants lower NMSE was consistently maintained with increased sampling (Figure. 4).

As an additional comparison, we defined the number of stimuli needed to produce an NMSE below 0.1 as a reasonable standard at which to compare. Among all methods WGPE, GPRS, and USRG methods required the fewest number of stimuli (GM: 122.4±27.5, 140.4±24.5, and 184.0±36.96 respectively) to reach this threshold.

**Fig. 5.**
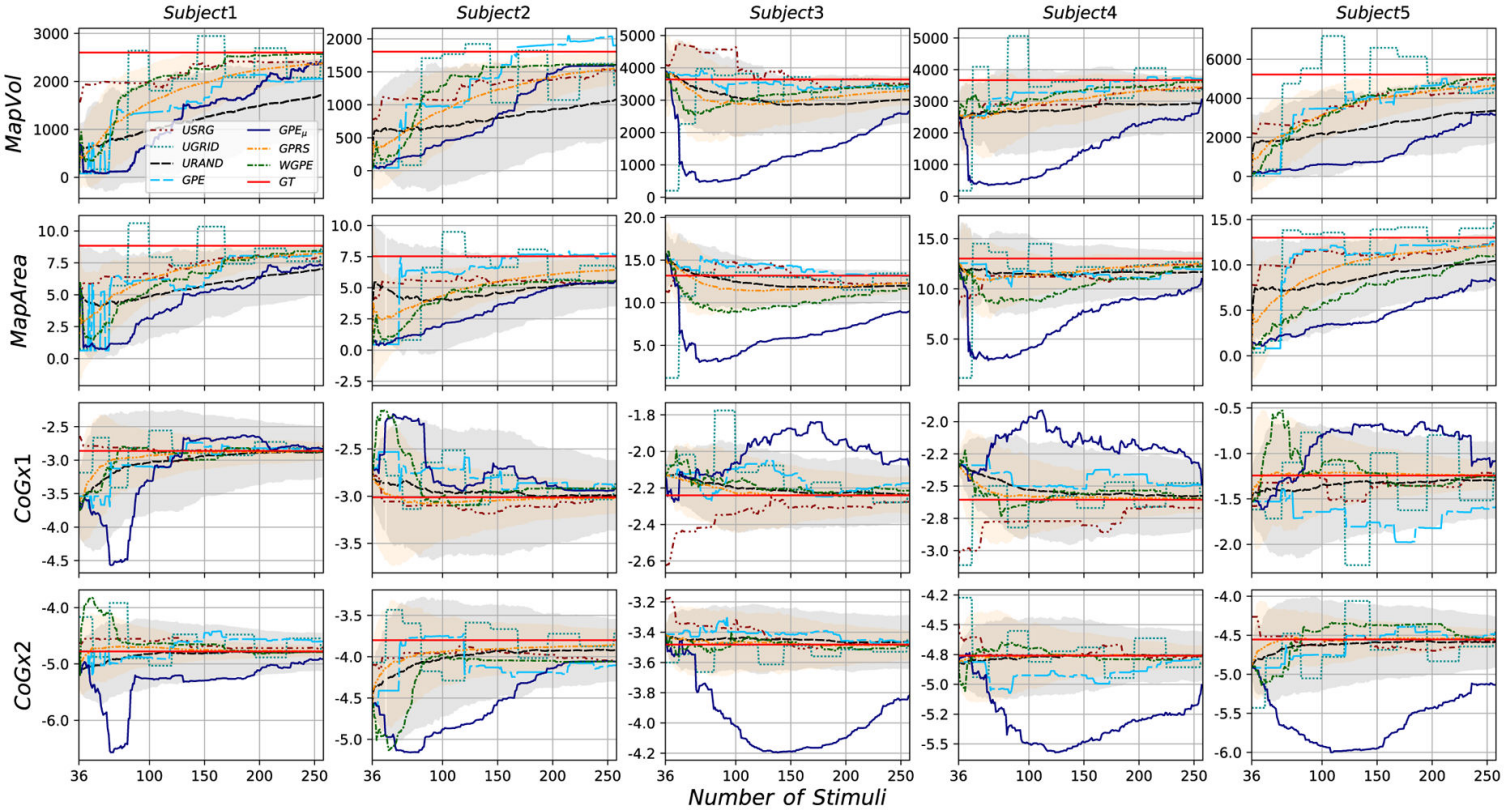
Map features evolution for all subjects and all methods. The ground truth’s values are represented by the red constant lines.

Evaluating results in terms of map features rather than NMSE, in Figure 5 we report results for those features for all subjects and all methods as a function of increasing number of stimuli, where the straight red lines in the plots show the GT value for each feature. Visual inspection of these figures suggests that some methods (e.g. UGRID and GPE_*μ*_) experienced high variability as the number of stimuli increased. USRG, WGPE, and GPRS typically were within 10% of the true value at full cardinality for all metrics across the subjects. UGRID, URAND, GPE, and GPE_*μ*_ all performed less accurately. For example, GPE_*μ*_ only reaches to within 15% and 28% of the true value across the features in subjects 3 and 5 respectively.

In addition to the results for the experimental methods, Table I also reports on results for the map features determined at full cardinality for the computational methods. In terms of the coefficient of variation (CV,^1^ relative standard deviation) for each feature, COGx1 and CoGx2 (CV = 0.038, and 0.0168 respectively) had the least variability across the different computational approaches in comparison to MapVol and MapArea (CV = 0.114, 0.0824 respectively). Across individuals MapArea results was consistent with USRG for the WGPE and GPRS methods, slightly larger for UGRID and GPE, and smaller for URAND and GPE*μ*. MapVol was observed to be consistent with USRG for WGPE, GPRS, UGRID, and GPE approaches while URAND and GPE*μ* approaches were characterized by smaller MapVol (see last row of Table I.

## VII. Discussion

As a first step towards highly efficient fully automated TMS mapping, in this study we described and compared several computational methods, motivated by the USRG approach (in which future stimulation loci are selected utilizing online feedback of resultant amplitude), for the selection of future stimuli. In terms of performance, among the experimental applied methods, the USRG sampling pattern was found to more rapidly reduce NMSE (relative to the GT) with added stimuli compared to RAND or GRID approaches, and resulted in the lowest NMSE at full cardinality for all subjects. The large reduction in NMSE in the first 50 stimuli is indicative of the active selection process made by the operator to maximize information using as few stimuli as possible. Using a pseudo-random walk pattern, van de Ruit and colleagues reported the production of maps with high correlation to the 100 stimuli full map in as few as 50 stimuli [9] (GM 63 stimuli). However, the use of at least 80 stimuli was recommended in order to generate a random sampling distribution that adequately covered the response area and maps that were less susceptible to test-retest variability. Our results showing greater efficiency (reduction of NMSE with fewer stimuli) of the USRG approach compared to a random approach confirms the suggestion in [9]. However, our random procedure was different than the one used by van de Ruit *et al.* [9]. In this study, 184 stimuli, representing 20% of the number of stimuli used to define the GT, were needed to meet our accuracy requirements, possibly indicating better performance than shown in van de Ruit *et al.* [9]. However, comparisons between our study and van de Ruit *et al.* [9] of the total number of stimuli needed to generate an accurate map are difficult, given differences in the total number of stimuli used to construct the GT and differences in the metric used to evaluate accuracy. Interestingly, results showed relatively small improvements in NMSE with increased stimuli in the GRID condition. While this may seem surprising, a recent investigation reported that only two stimuli per grid site were needed to create maps of equal validity to those obtained when five stimuli per site were used [10], consistent with our results.

The USRG approach can be characterized as a balance between exploitation of current knowledge about the excitable area and exploration of areas where excitability is uncertain. The relative success or failure of our computational approaches appears to be based on how each approach handled this exploration vs exploitation tradeoff. Among all computational methods tested, the GPRS and WGPE approaches had similar performance to the USRG method although they handle exploration-exploitation trade-off efficiently in two very distinct ways. For GPRS, exploration is introduced by the random nature of the algorithm while exploitation is introduced as the evolution of the GP mean modifies the probability of sampling regions of the space as new samples are obtained. For WGPE, exploration-exploitation is a consequence of the quantity (20) that scales the latent GP variance with the square of its mean, thereby increasing exploitation as the mean function evolves over time. These features underlie our confidence that WGPE and GPRS are the best strategies among the algorithms discussed in this study. On the other hand, the UGRID, URAND, and GPE methods all tend to place samples evenly in the space, neglecting exploitation.

As stated before, all the computational methods except URAND and GPRS are grid-based, meaning the algorithm selects the next stimulus from a finite grid (selectable set) without replacement. In our experiments, we used a finite 2D grid with 2mm resolution as our selectable set, based on the limitations of the coil tracking software. However, we noticed that these approaches were influenced by the preallocated grid. This mostly affected methods based on both GP spatial uncertainty and MEP amplitude like WGPE and GPE_*μ*_. Indeed GPE_*μ*_ was heavily affected by this factor, because it focuses only on the variations of MEP values (exploitation) and completely neglects spatial exploration. This is illustrated by comparing the GPE_*μ*_ results of this paper, in which a 2 mm selectable grid was used, to those of our previous paper [16] in which a 3 mm selectable grid was used. With a sparser grid, the algorithm is forced to select loci that are farther apart from each other (at the expense of losing definition of sharp peaks), whereas with the denser grid used here it got stuck exploiting small regions. This behaviour can be seen in Figure 3 where the loci selected by GPE_*μ*_ are overly concentrated in part of the ROI while ignoring unexplored regions.

### A. Limitations

A number of limitations may have impacted the findings of this investigation. A canonical brain was used for neuronavigated TMS instead of a reconstruction from the participants MRI. This may have impacted the TMS operator in the USRG condition, who may have used the subject-specific anatomy to guide coil position early during mapping. All experiments were conducted by an experienced TMS operator. It is unknown if the same result would be found if the individual selecting the stimulation sites was naïve to TMS mapping. This should be the subject of future investigation, as well as the inter-operator reliability. Only a single muscle was mapped. It is unknown whether multi-muscle mapping would be improved by a user-guided approach. To maximize such an approach, the TMS operator would need to be given feedback from each muscle collected. In this case, an automated or even random approach may better capture multiple representations at once. The coil orientation was fixed at 45° to midline (approximately perpendicular to the central sulcus) and was not adjusted for sulcus alignment on each individual stimulation. The method used to determine resting motor threshold was a less common alternative to the more common Rossini-Rothwell method [45] (which uses 5 of 10 stimulation greater than 50 *μ*V) as a threshold. Given maps for each participant were conducted with the same coil orientation and intensity in the same session, we do not believe that the coil orientation or method to determine RMT had an impact on the active sampling results presented here. Incorporation of the coil orientation and intensity is an achievable goal for improvement to our algorithm. In the future we intend update our algorithms to operate directly on the induced electric field for individual head models thereby incorporating coil orientation and stimulus intensity as well as three dimensional individual anatomy.

## VIII. Conclusion

In this paper, we leveraged different GP-based active learning strategies to study possibilities for automated TMS mapping, and compared these methods with traditional strategies. Warped GP entropy-based sampling and GP random sampling methods were found to be the best candidates for a fully automated replacement for operator-dependent mapping. We note that our study of different active sampling methods has potential applications to a wide set of spatial prediction problems outside the scope of TMS mapping.

## Figures and Tables

**TABLE I:** Map Features of the Final Map Generated With Every Method for All Subjects. The Ground Truth Values Are Shown in the Column Labeled GT. The Columns Show Both Experimental (Left) and Computational (Right) Results. Units Are Given for S1 and Are Identical for Each Feature for the Other Subjects. The Group Mean and Standard Deviations for Volume and Area Are Shown at the Bottom

	MAP Features	GT	Experimental Methods			Computational Methods					
			USRG	GRID	RAND	UGRID	URAND	GPE	GPE_*μ*_	GPRS	WGPE
S1	Volume (*μ*Vcm^2^)	2603.48	2450.64	195.80	2294.27	2190.25	1751.59±774.48	2251.15	2550.75	2560.36±91.19	2573.36
	Area (cm^2^)	8.83	7.78	1.07	7.03	8.08	7.12±1.77	8.68	8.16	8.34±0.57	8.41
	CoGx1 (cm)	−2.86	−2.77	−2.99	−2.72	−2.80	−2.86±0.27	−2.96	−2.86	−2.86±0.05	−2.87
	CoGx2 (cm)	−4.78	−4.72	−4.62	−4.70	−4.70	−4.76±0.27	−4.64	−4.81	−4.77±0.05	−4.79
S2	Volume	1806.05	1541.48	625.29	1369.29	1655.72	1101.68±702.86	1899.03	1618.74	1657.39±82.48	1644.27
	Area	7.53	5.49	3.69	7.10	7.45	5.63±2.22	7.55	5.66	5.99±0.67	5.86
	CoGx1	−3.01	−2.98	−2.24	−3.04	−3.10	−2.98±0.38	−2.90	−2.93	−2.98±0.08	−2.95
	CoGx2	−3.79	−4.05	−4.10	−3.84	−3.92	−3.90±0.37	−4.00	−4.03	−3.95±0.17	−4.00
S3	Volume	3641.94	3518.31	1881.54	3405.88	3417.25	3093.78±636.23	3456.38	3080.61	3529.35±133.33	3504.24
	Area	13.17	12.52	8.75	12.37	12.18	12.08±1.37	12.99	10.12	12.26±0.88	11.86
	CoGx1	−2.24	−2.28	−2.28	−2.33	−2.25	−2.24±0.13	−2.20	−2.19	−2.23±0.06	−2.20
	CoGx2	−3.48	−3.46	−3.36	−3.40	−3.45	−3.48±0.14	−3.45	−3.69	−3.51±0.07	−3.48
S4	Volume	3671.18	3442.27	1376.30	3021.00	3378.83	2966.97±889.25	3791.60	3554.99	3623.41±82.16	3611.56
	Area	13.03	12.48	6.68	12.25	12.31	11.53±1.98	11.79	12.13	12.47±0.67	12.24
	CoGx1	−2.61	−2.68	−2.42	−2.61	−2.61	−2.60±0.24	−2.51	−2.51	−2.59±0.04	−2.60
	CoGx2	−4.76	−4.74	−4.33	−4.72	−4.82	−4.75±0.23	−4.75	−4.83	−4.75±0.04	−4.76
S5	Volume	5211.60	4998.68	422.23	3640.00	4661.77	3345.79±1656.68	4456.19	3749.20	5004.62±227.48	5148.00
	Area	13.00	12.62	1.92	11.14	12.71	10.57±2.60	12.68	8.75	11.47±1.00	12.24
	CoGx1	−1.24	−1.20	−2.12	−1.17	−1.12	−1.24±0.39	−1.45	−0.98	−1.24±0.08	−1.25
	CoGx2	−4.55	−4.60	−4.02	−4.57	−4.67	−4.58±0.27	−4.60	−4.98	−4.55±0.10	−4.56
Group Mean	Volume	-	3190.28±1158.19	900.23±2.42	2746.09±825.80	3060.76±1051.04	2451.96±870.61	3170.87±957.32	2910.86±767.56	3275.03±1122.95	3296.29±1168.18
	Area	-	10.18±2.98	4.42±2.89	9.98±2.42	10.54±2.28	9.38±2.55	10.73±2.20	8.96±2.14	10.10±2.54	10.12±2.57

## Footnotes

1 *CV = σ/μ,* where *σ* is the standard deviation and *μ* is the mean of population.
